# Roles of the RANKL–RANK Axis in Immunity—Implications for Pathogenesis and Treatment of Bone Metastasis

**DOI:** 10.3389/fimmu.2022.824117

**Published:** 2022-03-21

**Authors:** Bo Li, Pengru Wang, Jian Jiao, Haifeng Wei, Wei Xu, Pingting Zhou

**Affiliations:** ^1^ Department of Radiation Oncology, Shanghai Ninth People's Hospital, Shanghai Jiaotong University School of Medicine, Shanghai, China; ^2^ Department of Orthopedic Oncology, Changzheng Hospital, Second Military Medical University, Shanghai, China

**Keywords:** bone metastasis, osteoclasts, osteoblast, RANKL/RANK, immune cells, denosumab

## Abstract

A substantial amount patients with cancer will develop bone metastases, with 70% of metastatic prostate and breast cancer patients harboring bone metastasis. Despite advancements in systemic therapies for advanced cancer, survival remains poor for those with bone metastases. The interaction between bone cells and the immune system contributes to a better understanding of the role that the immune system plays in the bone metastasis of cancer. The immune and bone systems share various molecules, including transcription factors, signaling molecules, and membrane receptors, which can stimulate the differentiation and activation of bone‐resorbing osteoclasts. The process of cancer metastasis to bone, which deregulates bone turnover and results in bone loss and skeletal-related events (SREs), is also controlled by primary cancer-related factors that modulate the intratumoral microenvironment as well as cellular immune process. The nuclear factor kappa B ligand (RANKL) and the receptor activator of nuclear factor kappa B (RANK) are key regulators of osteoclast development, bone metabolism, lymph node development, and T-cell/dendritic cell communication. RANKL is an osteoclastogenic cytokine that links the bone and the immune system. In this review, we highlight the role of RANKL and RANK in the immune microenvironment and bone metastases and review data on the role of the regulatory mechanism of immunity in bone metastases, which could be verified through clinical efficacy of RANKL inhibitors for cancer patients with bone metastases. With the discovery of the specific role of RANK signaling in osteoclastogenesis, the humanized monoclonal antibody against RANKL, such as denosumab, was available to prevent bone loss, SREs, and bone metastases, providing a unique opportunity to target RANKL/RANK as a future strategy to prevent bone metastases.

## Introduction

Metastasis is a complicated, multistep process that is responsible for roughly 90% of cancer-related deaths (1). Bone metastases are common in patients with solid tumors (especially for prostate, breast, lung, and kidney cancers), and the spine is the most significant site of advanced disease (2), whereas lesions in flat and appendicular bones are infrequent. For example, up to 50% of patients diagnosed with lung cancer will develop spinal metastases (3). This is partially explained by the ease of access to vertebral bodies in the thoracic and lumbar spine through the plexus vertebral system (4, 5) and the high bone marrow flow of some skeletal elements (6). Bone metastatic cancer can be extremely debilitating and is associated with devastating clinical complications, such as severe pain and skeletal-related events (SREs) including pathological fractures, spinal cord and nerve compression, hypercalcemia, and bone marrow aplasia, which result in a poor prognosis (7). As the mechanisms and pharmacological treatment of bone metastases have been increasingly recognized, growing evidence suggests that the bone microenvironment and the immune system may contribute to cancer bone tropism.

Normal bone development and maintenance are controlled by a balance in normal bone remodeling through bone-forming osteoblasts and bone-resorbing osteoclasts (8–10). In patients with cancer metastatic to the bone, osteolysis bone lesions often develop because of the tumor-induced ability to exacerbate osteoclastic activity, leading to imbalanced osteoclast and osteoblast functions (11). It has been traditionally thought that certain types of solid tumors that metastasize to the bone are characterized by disruption in the homeostasis of osteoblasts or osteoclasts, being commonly both damaged in most solid tumors (2). Along with an increased understanding of the interplay among dormant tumor cells, the bone microenvironment, and the bone marrow, it has been shown that the complex interactions in the bone marrow microenvironment are crucial for the initiation and promotion of metastases in the bone (12–14). More recent work has revealed that the bone itself also has a unique local immune environment favoring bone metastasis, arising from diverse primary tumor types (15). Reports show that a tumor can systemically produce hormones such as parathyroid hormone-related protein (PTHrP) mediating osteoclastogenesis by enhancing the expression of the receptor activator of nuclear factor kappa B ligand (RANKL) (16). RANKL and RANK, a receptor–ligand pair of the TNF receptor superfamily, can regulate osteoclast development and bone metabolism (17, 18). In addition to the critical function of RANKL and RANK in normal bone resorption and remodeling, the RANKL/RANK pathway also controls many other physiological processes such as immunity, the proliferation and division of mammary epithelial cells, as well as mammary gland formation during lactation (19).

Osteoclasts and osteoclast precursors express RANK, whereas RANKL is mainly produced by osteoblastic lineage cells (20), bone-marrow stromal cells, immune cells (20–22), and some cancer cells (23). The binding of RANKL to RANK is an essential mediator of differentiation and osteoresorption function of the osteoclast, leading to bone resorption. Moreover, RANKL expression is crucial for the immune system, as it serves as an important molecule in optimal T-cell activation, mediates dendritic cell survival, and regulates the development of lymph nodes and Peyer’s patches (24, 25). The significance of the contribution of RANKL/RANK in the bone microenvironment and the immune system during bone metastasis process strengthens the notion that abrogation of RANK/RANKL signaling represents a key therapeutic target for cancer therapy. This review summarizes the current state and progress of the bone microenvironment and the immune system in the carcinogenesis process, which has propelled a deeper understanding of bone metastases. Unveiling the potential capacity of the RANKL/RANK axis to modulate the tumor immune microenvironment opens the door to the development of pharmacological inhibitors of RANKL as a potential therapy for bone disease in cancer patients.

## Immune System in Cancer

Dating back to 1889, Stephen Paget postulated the idea of metastatic spread, that primary tumors induce changes to distant organs, due to the high degree of crosstalk between cancer cells and their microenvironment (26). The immune microenvironment, wherein the immune cells are primarily located in or around the cancer and the adjacent lymphoid tissues, plays a vital role in metastatic cascade in cancer (27, 28). Primary tumors can influence the systemic and local immune homeostasis, promoting chronic inflammation and consequently suppressing immune activity that facilitates immune evasion and metastasis formation. These immunosuppressive cell populations promoting metastasis include macrophages (29), conventional CD11b+Ly6G+ neutrophils (30, 31), platelets (32), as well as myeloid-derived suppressor cells (MDSCs) (33). Conversely, CD4+ T cells and CD8+ cytotoxic T lymphocytes (CTLs) exert antimetastatic effects by activating adaptive immunity to control primary tumors (34, 35). It is deemed that CD4+ T cells can either promote or prevent tumor progression. Regulatory T (Treg) and T helper 17 (Th17) CD4+ T-cell subsets have emerged as key elements facilitating a pro-tumor inflammation environment that could favor cancer initiation, progression, and metastasis (36). Several cytokines, such as RANKL, produced by CD4+ T cells, reportedly promote breast cancer progression and metastasis (37). The maturation and antigen presentation of dendritic cells (DCs) play diverse roles in the protective antitumor response (38, 39). CD8+ CTLs can recognize specific antigen bound to major histocompatibility complex (MHC)-I on DC, then they were activated and can produce interferon γ (IFNγ), perforin, and granzyme B that contribute to tumor cell cytolysis (40). Together, these results reveal that therapeutic strategies that can play roles in regulating immune responses may be a feasible immunotherapeutic action for patients with metastatic cancers.

## Immune System in Bone Metastasis

Bone metastases have traditionally included osteolytic and osteoblastic metastases classified according to the predominance of lysis or sclerosis in the bone. Growing evidence has suggested the fact that mixed-type bone metastases containing both osteolytic and osteoblastic change were also observed in most patients (41). Bone metastases in breast cancer patients are dominantly mediated by osteoclast-induced osteolytic lesions; local bone forming and osteoblastic lesions are also observed (2). Bone metastases in many patients with lung cancer or multiple myeloma are preponderantly osteolytic, leading to the occurrence of focal bone destruction (42). Conversely, in the case of prostate cancer, the bone appears as dense osteosclerotic lesions and is characterized by increased osteoblast activity (43). The mechanism of bone metastases is complex; tumor cells, osteoblasts, osteoclasts, and the mineralized bone matrix cooperate to promote bone metastasis (**Table 1**) (44–58, 60, 63–65). In osteolytic bone metastases, multiple cytokines and growth factors secreted by tumor cells, such as PTHrP, RANKL, interleukins, prostaglandin E, tumor necrosis factor (TNF), and macrophage colony stimulating factor (MCSF), are responsible for hyperactive osteoclast activity, leading to osteoclast bone resorption and promoting osteolytic metastasis (59, 61, 62). In turn, osteoclastic bone resorption releases growth factors, such as TGFβ, IGFs, PDGFs, and BMPs, to promote cancer proliferation, sequentially further enhancing the secretion of osteolytic factors and driving a feedforward to fuel tumor growth in the bone (66, 67). In osteoblastic metastases, studies have established the scientific foundation for the vicious cycle of tumor cells and osteoblasts. Tumor cells produce factors, including FGFs, urokinase-type plasminogen activator (uPA), endothelin-1 (ET-1), prostate-specific antigen (PSA), insulin-like growth factors (IGFs), bone morphogenic proteins (BMPs), and vascular endothelial growth factor (VEGF), which stimulate osteoblast proliferation and differentiation (68, 69). This process, in turn, stimulates prostate cancer growth and invasion (70). Additionally, studies have also implicated the importance of several intracellular pathways, such as mitogen-activated protein kinase (MAPK), nitric oxide (NO), and Wnt signaling, in bone-forming osteoblasts (71, 72).

**Table 1:** Regulating factors associated with the bone metastasis process.

Regulating factors	Action mechanism	References
**Osteoclastic metastases**		
RANKL/RANK	Regulate osteoclast differentiation, activity, and survival	(44)
PTHrP	Activates the bone resorption activity of osteoclasts	(45)
OPG	Disrupts osteoclastogenesis and subsequent bone resorption by enhancing the local production of RANKL	(46)
TNF-α	Upregulates the expression of RANKL and induces osteoclast differentiation	(47)
IL-11	Directly or indirectly promotes osteoclastogenesis by enhancing the production of RANKL	(48)
IL-6	Promotes osteoclastogenesis *via* interaction with the IL-6 receptor to induce RANKL expression in osteoblasts and stromal cells	(49)
IL-7	Induce osteoclastogenesis *via* STAT5 signaling	(50)
GM-CSF	Stimulates osteoclastogenesis *via* Ras/ERK signaling	(51)
IL-8	Stimulates osteoclastogenesis and bone destruction in metastatic bone diseases	(52)
PGE	Promotes the production of IL-11 by osteoblasts	(53)
Jagged 1	Promotes fusion of osteoclast precursor cells by directly binding to monocytes	(54)
CTGF	Stimulates angiogenesis, which helps tumor growth and osteoblast proliferation	(55)
HIF1α	Induces osteogenic inhibitory factors overexpressed, which strongly correlate with osteolytic bone destruction	(56)
**Osteoblastic metastasis**		
ET-1	Suppresses osteoblast apoptosis by stimulating the calcineurin/NFAT pathway	(57)
PSA	Stimulates osteoblasts by preventing IGF from its binding protein	(58)
BMP	Promote osteoblast differentiation	(59)
uPA	Stimulates the osteoblasts	(60)
DKK-1	Converts osteolytic metastasis to osteoblastic metastasis	(61)
PTHrP	Facilitates osteoblastic alterations	(62)

**Figure 1:**
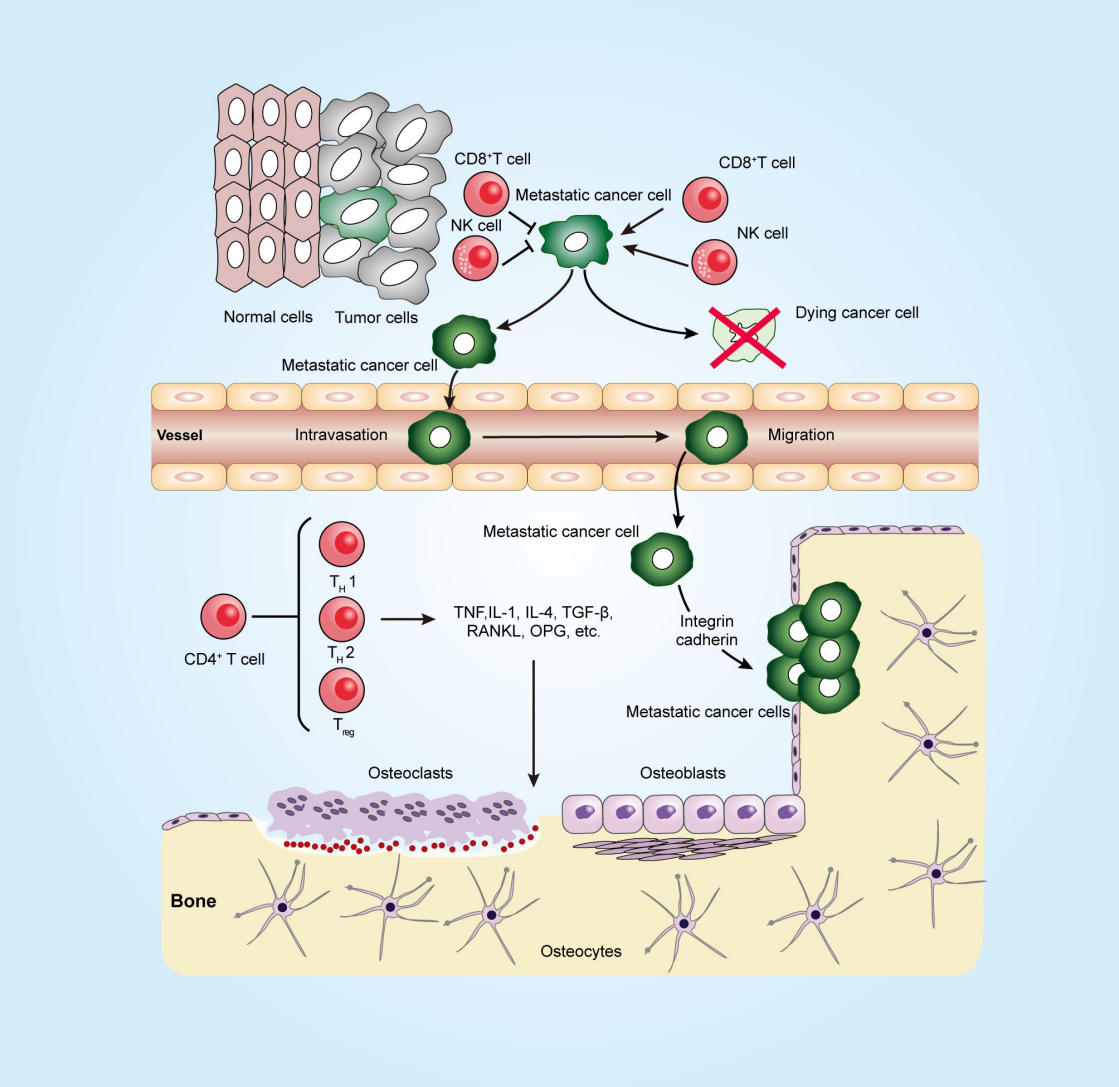
Immune system in a long journey to develop bone metastasis. To establish the metastatic tumor, cancer cells escape from the tumoricidal immune response that is mediated by killer cells, such as CD8+ T cells and natural killer (NK) cells, and then invade through the surrounding stroma and intrude into blood vessels (intravasation). At the metastatic site, the arrested tumor cells in microvessels escape from the blood vessel (extravasation). Metastatic tumor cells can interact with both osteoclasts and osteoblasts in the bone microenvironment and release factors, such as TNF, TGF-β, and RANKL, to promote osteoclastogenesis and osteoblastogenesis. CD4+ T cells can produce not only osteoclastogenic cytokines such as IL17 and TNF but also anti-osteoclastogenic cytokines such as IFNγ and IL4. The figure was designed using Adobe Illustrator CC.

Owing to the close connection between the immune system and the bone in many diseases, such as inflammatory diseases and postmenopausal osteoporosis, we focus on how the two systems affect each other. Evidence is accumulating to support many shared regulatory factors, including cytokines, receptors, signaling molecules, and transcription factors, between the immune and bone systems (73, 74) (**Figure 1**). Soluble factors secreted from antigen-stimulated immune cells, such as interleukin-1 (IL-1) and RANKL, have a central role in activating osteoclast (75). It has been demonstrated that activated T lymphocytes might have the capacity to induce an osteoclastic phenotype by directly acting on osteoclast-precursor cells (76, 77). It was also observed that mice lacking cytotoxic T lymphocyte protein 4 (CTLA4) with systemically activated T lymphocytes trigger an increased osteoclast number response and induce an osteoporotic phenotype (78). However, not all T-cell responses associated with bone loss have such a deleterious outcome. The osteoprotegerin (OPG), IFNγ, interleukin (IL)-4, and transforming growth factor-β (TGF-β) produced by T cells appear to prevent T lymphocyte-mediated osteoclastogenesis (79, 80). In addition, Th1, Th2, and CD8+ T cells contribute to the repression of, other than the stimulation of, osteoclastogenesis in murine (81–85), and this blocking effect on murine and human osteoclast differentiation was observed in Treg cells (81). Arthritis was observed after the suppression of Treg cells, whereas bone destruction was ameliorated after the transfer of Treg cells into T-cell-deficient mice, suggesting that Treg cells are potentially beneficial in the bone protective effect (86, 87). The activated T cells showing pro-osteoclastogenic or anti-osteoclastogenic ability are usually thought to potentially depend on the local environment. New data have indicated the involvement of other immune cell types, such as DC, neutrophils, and natural killer cells, in increased osteoclastogenesis in diseases. For instance, DCs can undergo transdifferentiation into the osteoclast cell type under inflammatory conditions (88).

More findings provide evidence that the immune system performs a critical role in the progression and the treatment resistance of tumors. It is known that a complex interplay of immunity in primary tumors can have a profound impact on the development of tumors (89, 90). Here, we focus on the contributions of the immune microenvironment to the bone metastasis of primary cancer. In this contribution, the multiple inhibitory and stimulatory effects of the immune system on host cells within the bone microenvironment were systematically reviewed, emphasizing the critical roles of immunity in the modulation of bone metastases (91). Partially the regression of tumor bone metastasis is due to the antagonistic effect of several programs, such as natural killer (NK) cell- or effector T cell-mediated lysis. However the majority of immune cells, such as regulatory T cells (Treg), dendritic cells (DCs), MDSCs, and macrophages, serve as a phenotype promoting cancer bone metastasis. Immunosuppressive myeloid-derived suppressor cells infiltrate in tumors driving tumor growth, sensed by inhibiting the host antitumor immune responses in the tumor. In bone metastasis, signals derived from the tumor and the microenvironment allow myeloid-derived suppressor cells to act as progenitors of the osteoclast to enhance tumor-induced osteolysis (92, 93). The impact of T cells on bone metastasis has also been recognized. Treg cells, which maintain immune homeostasis through impeding aberrant immune response against self-antigens, also suppress antitumor immune response (94–96). A study found that a dose-dependent increase in the recruitment of Treg cells in a mouse model of breast cancer treated with PGE2 derived from tumor leads to increases in the cellular apoptosis of CD8+ T cells and promotes bone metastasis (97).

## The Crucial Role of RANKL/Rank Signaling in Bone Remodeling

RANK was first identified in 1997 *via* expression cloning techniques in the course of studying a novel tumor necrosis factor receptor (TNFR) superfamily member. RANKL, also known as TNFSF11 (tumor necrosis factor receptor super family 11), TRANCE (TNF-related activation-induced cytokine), OPGL (osteoprotegerin ligand), and ODF (osteoclast differentiation factor), was characterized as the cognate ligand of TNFR superfamily member (21). Over 20 years, much has been researched about the important biology of TNFSF, their receptors and the intracellular signaling pathways in patients with autoimmune or inflammatory disorders. TNFSF and TNFR members are broadly expressed in immune cells including macrophages and brain glial cells and are usually related to the expression of immune system cells (98). TNFSF proteins perform immunoregulatory functions through displaying a lymphoid tissue organizer and lymphocyte stimulation activity and increasing the survival and function of lymphocytes (99–103). Moreover, TNFSF is closely associated with neuronal activity, such as CNS injury and neurodegenerative diseases (104). Recent evidence indicates that TNFSF is one of the major mechanisms of the development and survival of osteoclasts (103). These scientific discoveries have led to the identification of novel therapeutic options, through inhibiting or activating TNFSF signaling pathways, to control diseases.

The TNF superfamily of cytokine-like molecules included a superfamily of 19 ligands, while the TNFR superfamily consisted of 29 proteins acting as receptors of these ligands (105). One important example is the system consisting of RANKL and its signaling receptor RANK. In 2016, a study reported that leucine-rich repeat-containing G-protein-coupled receptor 4 (LGR4, also called GPR48) is another receptor for RANKL. The findings suggest that LGR4 competes with RANK for RANKL binding in osteoclasts, and a negative regulatory feedback loop of LGR4 has been unveiled to restrict RANKL osteoclastogenesis through multiple mechanisms (106). On the other hand, further research about the molecular mechanisms for the crucial roles of LGR4 mediation of RANKL signaling in many other processes has not appeared. The gene TNFSF11, located on human chromosome 13q14 and a conserved syntenic region on mouse chromosome 14, encoded the RANKL protein (107, 108). RANKL was initially identified on murine thymoma cell line EL40.5, and then in 1997, functional genomics and biochemical analyses identified RANKL, expressed by activated T cells, as a stimulator of the DCs (109), and RANKL played an essential role in regulating the DCs–T cells interaction (110). RANKL is a molecule acting as a three-part complex, containing a small N-terminal intracellular domain, a transmembrane segment, and a c-terminal extracellular domain consisting of a stalk and a receptor-binding region (108).On the other hand, RANKL exists in soluble forms upon proteolytic cleavage of the extracellular stalk region by matrix metalloproteinases (MMPs) such as MMP-14 (111, 112). However, RANKL as a membrane-anchored molecule functions significantly more efficiently (113). RANKL mediated oligomerization of the receptor RANK on cells, and it activated RANKL/RANK signaling and downstream responses. The activation of the RANKL/RANK pathway initiates a cascade of intracellular signaling molecules such as tumor necrosis factor receptor-associated factor (TRAF) family members, which can culminate in nuclear factor κB (NFκB), AP-1, and NFATc1 signaling activation that are responsible for osteoclast differentiation and function (114, 115). By contrast, it is reported that RANK can activate osteoclasts independent of ligand through receptor self-association (116). However, most physiological functions of RANK are generally deemed to be ligand-dependent to be the starting point of the RANKL/RANK signaling cascade (117).

The bone is a dynamic tissue that consists of osteoblasts and is resorbed by osteoclasts in the bone remodeling cycle, which is responsive to the skeleton for optimal function. Once a bone matrix of osteoclasts, most osteoblasts will undergo apoptosis, then a proportion of cells will differentiate into osteocytes, which make up the majority of bone cells in the adult skeleton (118). Of note, osteocytes are reprogrammed from differentiated osteoblast cells, and the functions of osteocytes are still being investigated. Although it appears that osteocytes do not express RANKL mRNA (119), data from previous study suggest that osteocytes could regulate RANKL expression through other bone cells. Osteocytes, based on the location within the bone matrix and cellular morphology, could regulate bone remodeling through modulating the production of RANKL (112, 120). The resorptive effect reflects the important role of RANKL on osteoclasts and/or their precursors (121). Studies in mice lacking TNFSF11 or TNFRSF11a gene have illuminated the cellular and molecular mechanisms by which RANKL controls tooth eruption owing to the function of RANKL in osteoclast development and bone metabolism (122, 123). A RANKL transgenic mice model greatly reduced bone mass and increased the number and enhanced the activity of osteoclasts, demonstrating the systemic nature of the increased bone turnover (124). Binding of RANKL to its receptor RANK, located on the surface of pre-osteoclasts and mature osteoclast cells, triggers pre-osteoclast differentiation into multinucleated, fully functional osteoclasts (125). A fine balance of osteoclasts and osteoblasts is tightly related to the RANKL–RANK system because mice deficient in either RANK or RANKL show a complete absence of osteoclasts and develop severe osteopetrosis (126, 127). It has also been reported that RANKL inhibition in osteocyte-specific RANKL-deficient mice was effective to protect against bone loss and deletion of osteocyte RANKL, conferring an increased cancellous bone mass in osteogenesis imperfecta mice (128, 129). It was also suggested that vesicular RANK, which is secreted from the maturing osteoclasts, binds osteoblastic RANKL and promotes bone formation by triggering RANKL reverse signaling (130). These results suggest that osteocyte RANKL, whether in healthy or diseased individuals, greatly affects bone resorption, and targeting RANKL can be classified as appropriate for the development of drugs.

## RANKL–Rank Axis in Immunity

It has become clear that cells of both innate and adaptive immunity are able to respond to tumors and promote the effective elimination of cancer cells, which ultimately help fuel tumor progression. With the crucial role for driving either co-stimulation or co-inhibition of the immune response through various mechanisms, TNFSF members provide unique biophysical and biochemical cues to control both innate and adaptive immunity (105). Efforts on study about the RANKL–RANK pathway showed that RANKL provided by T cells following antigen receptor stimulation can significantly stimulate the survival and function of DCs, chief inducers of adaptive immune response across the body (107, 131, 132). The RANKL/RANK pathway not only stimulates immune the system (e.g., lymph-node, B-, and T-cell development) but also inhibits the immune system (e.g., generation of regulatory T cells and induction of T-cell tolerance) (133, 134).

Although a rare population of DCs exists among the immune cells within tumors and lymphoid organs, DCs are central to adaptive immune response, acting to initiate the antigen-specific immunity and tolerance during invasive microorganisms (135, 136). Consequently, multiple clinical strategies, including harnessing the activity of DCs and the generation of DC-based vaccines, are being detected to target DCs for cancer treatment. Findings reveal the mechanisms of DCs in the activation of immunity that rely not only on capturing antigens and presenting them to T cells in the lymph nodes but also on providing immunomodulatory signals through cell–cell contacts and cytokines (137, 138). DCs initiate CD8+ or CD4+ T-cell activation by antigen-loaded MHC class I or II molecules, respectively expressed on the DC surface, and by providing potent co-stimulatory signals expressed by the T cells (139, 140). In fact, differentiation of naive CD4+ T cells into each T-cell subset, such as Th1, Th2, and Th17 cells, is dictated by DCs providing the additional activation and developmental signals (140). Importantly, the maintenance, homeostasis, maturation, and activation of DCs during an immune response are supported by TNFRSF members, such as RANK, CD40, and LTβR, that are strongly expressed on DCs (21, 141). The study explored that the role of the interaction between RANKL on the activated T cell and RANK on the activated DC in enhancing the survival of DC may be through the induction of Bcl-xL and the upregulation of CD40 (105). Transforming growth factor β-activated kinase 1 (TAK1), a mitogen-activated protein kinase, mediates signal transduction from CD40 and RANK. The result that reduced survival of TAK1-deficient DCs cannot be rescued with neither RANKL nor an anti-CD40 agonistic antibody clarified that RANKL plays essential roles in promoting DC survival in a TAK1-dependent manner (142).

RANKL can modulate immune responses, balancing inflammatory processes and immunosuppression. The DC survival promoted by RANKL raises the immune response and increases inflammation. It was found that RANKL signaling in DCs significantly upregulated in Fas-deficient strain MRL/lpr mice leads to a rapidly progressing autoimmunity, suggesting that the activation of RANKL signaling may be regulated by Fas-induced signaling (143). On the other hand, RANKL can regulate DC-mediated immunosuppression. The data demonstrate that RANKL expression induced on keratinocytes can stimulate a molecular pathway that couples the epidermis to local and systemic immunosuppression by regulating the function of epidermal DCs, which is crucial for the peripheral homeostasis of regulatory T cells (144). Thus, the RANKL–RANK interaction could selectively either promote or suppress immunity determined by the specific phase of the immunity cycle at which this pathway is activated (145).

Activated CD4+ and CD8+ T cells can produce RANKL, which is also expressed in lymph nodes, spleen, thymus, and immature CD4 CD8 thymocytes (146, 147). Emerging insights into how RANKL expression is influenced provide the view that calcineurin, extracellular regulated protein kinases (ERK1/ERK2), and protein kinase C (PKC)-regulated signaling pathways can regulate RANKL expression in T cells (148, 149). In addition to the role of T cells in RANKL, RANKL/RANK signaling plays a key role in T-cell physiology. RANKL and RANK knockout mice, which failed to develop lymph nodes, have been shown to have intact splenic architecture and Peyer’s patches (150, 151). In adjuvant arthritis, a T-cell-driven experimental arthritis, the immune response is potentially influenced by RANKL blockade (152). On the other hand, specific primary T-cell responses were increased following RANKL treatment. Interestingly, crucial memory responses were observed only in mice injected with RANKL-treated dendritic cells (132). RANKL inhibition *in vivo*, including the use of a soluble RANK-Fc molecule, does not prevent the priming of LCMV-specific T cells, but it has damaged effects on proliferation of CD4+ T cells to the viral antigen after a period of infection, reaching a point at which RANKL seems to play a role in memory T-cell responses (153). At the same time, data in lymphocytes suggest that decreasing RANKL expression does not help regulate the generation of cytotoxic T cells in normal lymph node condition (153).

The development of B cells relies on the production and regulation of chemical species, such as RANKL, OPG, IL-7, and C-X-C Motif Chemokine Ligand 12 (CXCL12) that are produced by bone marrow stromal cells and osteoblasts (154). RANKL or RANK deficiencies created an interference of B-cell development due to the loss of the B-cell development environment. However, a study revealed that neither obvious loss of B-cell development in the bone marrow nor functions such as immunoglobulin secretion were observed in RANK-depleted mice, suggesting that the normal development and homeostasis of B cells do not require B-cell intrinsic RANK expression (155). Thus, these results showed that the effect of RANKL in B-cell development was caused by the interaction between RANKL and an alternative receptor.

## RANKL–Rank Axis in Bone Metastasis

**Figure 2:**
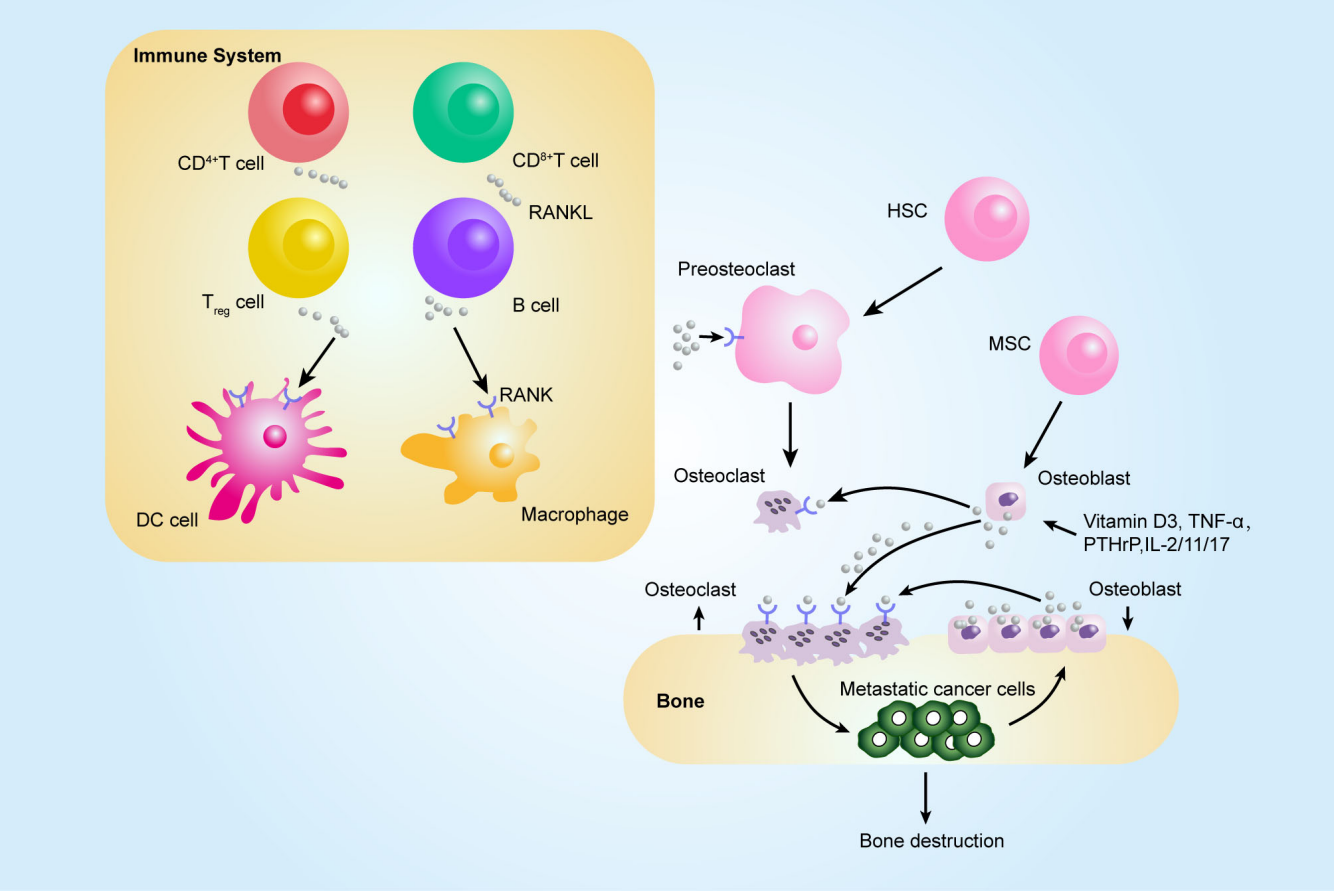
Contribution of the RANKL–RANK axis in immune system and bone metastasis. Among tumor-infiltrating immune cells, the expression of RANKL has been observed on all immune cell types, such CD8+ T, CD4+ T, and Treg cells, and RANKL can act on dendritic cells (DCs) to promote their survival and to prolong T–DC interactions. Bone-resorbing factors, such as vitamin D3, PTHrP, IL-1, IL-11, IL-17, and TNF-α, act on osteoblasts to induce RANKL, which binds to RANK present at the surface of osteoclast progenitors (pre-osteoclasts), which results in bone resorption by mature osteoclasts. The figure was designed using Adobe Illustrator CC.

Although RANK expression was primarily found in osteoclasts and their progenitors, recent data also indicate that RANK is expressed on tumor cells, appearing to regulate metastases from establishing tumors (156). The role of RANKL/RANK in immune and bones is as essential for autoimmune diseases affecting the bone as the development of bone metastasis from tumors. Causes of RANKL/RANK in bone metastasis could be divided into those that enhance osteolysis and those that have an effect on promoting metastasis (**Figure 2**).

RANKL was found to exert promigratory effects in a breast cancer model and to promote their metastasis to the bone *via* PTHrP produced by breast cancer cells (157). Recent investigations have shown that osteolytic bone metastases through osteoclast activation may be the important function of RANKL/RANK in tumor bone metastasis. Moreover, this pathway also contributes to tumor growth and metastasis in a bone-independent manner. The RANKL/RANK pathway is an important checkpoint that influences the antitumor immune response or directly affects bone metastasis (158). It was demonstrated that hormone receptor-negative breast cancer patients with higher RANK expression are at a higher risk of bone metastasis and have worse recurrence-free survival (159). It was also suggested that RANKL, with chemokine function for recruiting cancer cells to the bone, can stimulate the migration of breast cancer cells to the bone (156). With respect to the role of RANKL outside of the bone in triggering metastasis, one study showed that infiltrating Tregs expression of RANKL can drive the metastasis of breast cancer through altering the secretion of inflammatory factors in metastasis (37). With the increased knowledge regarding the molecular pathways downstream of RANKL/RANK signaling in bone metastasis, it is expected that RANK signaling has a close connection with metastatic potential through the activation of IKK-a (160).

Pro-osteoclastogenic factors, including parathyroid hormone (PTH), vitamin D3, TNF-α, Wnt 5A, and Sclerostin (SOST), can promote osteoclastogenesis through inducing RANKL expression in osteoblasts, stromal cells, or T cells (161–163). There are many additional osteotropic factors, including IL-1, IL-6, and IL-11, exerting osteoclastogenic activity by leading to RANKL expression in osteoblasts (164). Upon the phosphorylation of JNK, Wnt5a-Ror2 upregulates the expression of RANK in osteoclast precursors, c-Jun is recruited to the promoter of the RANK gene, and RANKL-induced osteoclastogenesis is activated (162, 165). RANKL levels can also influence the activation of osteoclast-specific genes (TRAP, cathepsin K, and MMP-9) (166).

Metalloprotease–disintegrin TNF-a convertase (TACE) (98), including MMP-7 and MMP-14, can convert the membrane-bound form of RANKL to a soluble form. In a MMP-7-deficient prostate cancer model, it is observed that MMP-7-deficient treatment reduces the risk of osteolytic bone metastasis as a result of aberrations in RANKL processing (167). However, the impact of circulating sRANKL in cancer biology is now in dispute. Increased serum sRANKL levels were observed in a cohort of 61 patients with lung or breast cancers with bone metastasis compared with healthy volunteers (168). In other studies, serum sRANKL levels between bone metastasis patients from prostate, lung, or breast cancer and those without bone metastasis did not discriminate (169–171).

## Development of RANKL Inhibitors

Building on these foundations about the roles of the RANKL/RANK pathway in the immune system, bone metastasis, and many other physiological processes such as the proliferation and division of mammary epithelial cells as well as the mammary gland formation during lactation (**Table 2**) (37, 172–179, 182, 183), researchers have exploited the inhibitors targeting this axis to control the primary tumor development, reduce bone metastasis, and even exert a direct antitumor effect *via* regulating antitumor immune responses (180, 181). Antibodies targeting RANKL were preferably used; this behavior might be attributable to an unintended receptor agonist with those targeting the receptor. Denosumab, a humanized monoclonal RANKL antibody, is one such candidate, which can play an important role in different physiological activity including the immune system, bone, or cancer (108). On target cells, denosumab can bind to the DE loop region of both soluble and membrane-bound RANKL to inhibit RANKL/RANK intracellular signaling. The DE loop region is one of the surface loop structures of human RANKL binding to and oligomerizing its receptor RANK on responding cells (184). In 2001, Amgen started studies about significant therapeutic and clinical safety of denosumab, which was applied for osteoporosis patients, bone destruction caused by advanced cancer, and bone loss generated by cancer treatment in early-stage cancer.

**Table 2:** Mice genetic phenotypes on the RANKL/RANK axis.

Phenotype	Tumor models	Key findings	References
Skeletal system	RANKL KO or RANK KO	Osteopetrosis, no tooth eruption	(172)
Immune system			
T lymphocytes	RANKL KO or RANK KO	CD4/CD8+ ratio normal, T-cell activation	(173)
B lymphocytes	RANKL KO or RANK KO	Development impaired	(173)
Thymus	RANKL KO or RANK KO	Size/development impaired, mTECs impaired (RANKL KO) Size/development normal, mTECs impaired(RANK KO)	(174)
Lymph nodes (LN)	RANKL KO or RANK KO	Peripheral LN defect, Peyer’s patch small	(175)
Spleen	RANKL KO or RANK KO	Normal architecture, extramedullary hematopoiesis	(176)
Dendritic cells	RANKL KO	No deficit in peripheral DCs and be limited to enhancing DC survival	(177)
Others			
Mammary gland	RANKL KO or RANK KO	Development impaired (RANKL KO) development impaired, inhibition of tumorigenesis (RANK KO)	(178)
Inflammation	RANKL KO or RANK KO	Osteopetrosis, no osteoclasts, no joint erosion	(175)
Anti-cancer			
Breast cancer	Orthotopic MT2 mammary carcinoma (Her-2 overexpression)	Reduced the frequency of spontaneous lung metastasis	(40)
Melanoma	B16 melanoma	Reduced the incidence of experimental lung metastasis	(179)
Colon carcinoma	CT26 colon carcinoma	Resulting in eradication of subcutaneous tumors in some models	(180)
Lung carcinoma	3LL lung carcinoma	Sequencing of antibodies: anti-PD-1 mAb before anti-RANKL mAb is superior to initial treatment with anti-RANKL mAb followed by anti-PD-1 mAb	(180)
Prostate carcinoma	TRAMP-C1 prostate carcinoma	Sequencing of antibodies: anti-PD-1 mAb before or concurrent with anti-RANKL mAb is superior to initial treatment with the anti-RANKL mAb	(181)

Osteoporosis is an increasingly common medical and socioeconomic matter characterized by decreased bone mass and strength, resulting in the propensity of fractures and substantial bone-associated morbidities. Denosumab, due to its role in osteoclastogenesis, is one of the most promising novel treatments of osteoporosis and has recently been approved in Europe and the US (185). The optimal denosumab dosage used in the treatment of osteoporosis turned out to be 60 mg at 6-month intervals subcutaneously. The antiresorptive effects of denosumab inhibiting the recruitment and activity of osteoclasts decline rapidly >6 months after each injection, and rapid decreases in bone mineral density (BMD) and bone resorption occur, losing fracture protection (186). Therefore, denosumab administration every 6 months is recommended after the last denosumab use to prevent a potential rebound effect (187). Very rare cases of osteonecrosis of the jaw and atypical femoral fractures occurred in osteoporosis cases under long-term denosumab exposure, but these possible side effects remain to be confirmed in long-term follow-up (188).

In a study including 252 postmenopausal women with early-stage breast cancer who receive endocrinotherapy with an aromatase inhibitor, after 24 months of follow-up, the result suggested that lumbar spine BMD in the denosumab group is 7.6% above the placebo group, and in the total hip, BMD is 4.7% above in denosumab individuals (189). Initiation of androgen-deprivation therapy (ADT) in localized prostate cancer patients is associated with rapidly decreased BMD and increased risk of fracture (190, 191). In a placebo-controlled trial in 1,468 nonmetastatic prostate cancer patients receiving ADT, a significant reduced incidence of new vertebral fractures was observed in the denosumab group and an increased BMD occurred, leading to significant differences in the denosumab and placebo groups (192).

**Figure 3:**
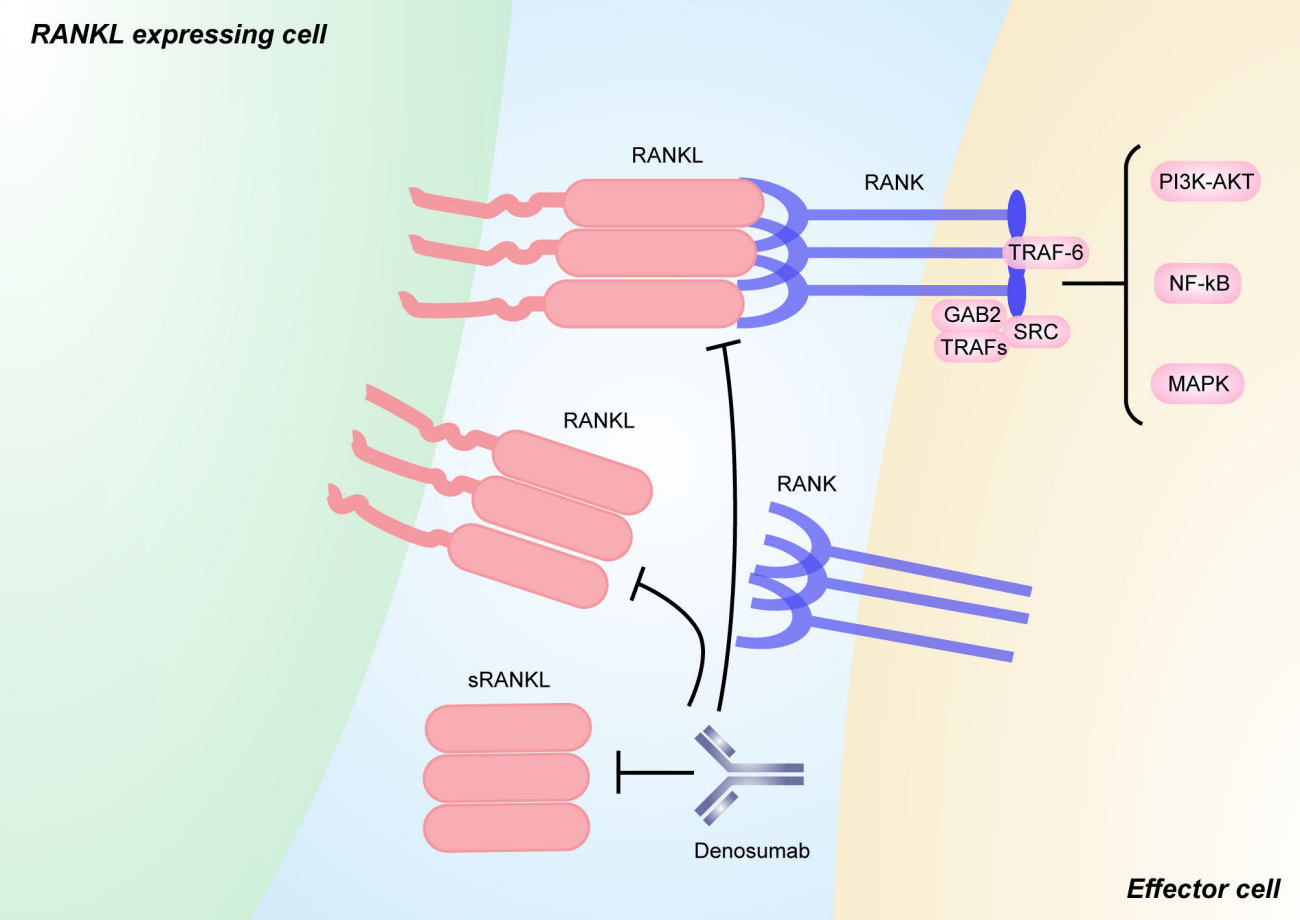
RANKL/RANK signaling and its inhibitors. RANKL is a type II transmembrane molecule that contains a small N-terminal intracellular domain, a transmembrane region, and a c-terminal extracellular domain consisting of a stalk and a receptor-binding region [111]. sRANKL is derived from the membrane-bound form through alternative splicing or proteolytic cleavage. Binding between RANKL and RANK induces the recruitment of TNF receptor associated (TRAF) proteins (including TRAF6), GRB-associated-binding protein 2 (GAB2), and SRC, which activates downstream signaling pathways, such as NF-κB, MAPK, and PI3K–AKT pathways. Pharmacological inhibitors targeting both soluble and membrane-bound forms of RANKL that have been used in humans include the anti-RANKL monoclonal antibody denosumab. The figure was designed using Adobe Illustrator CC.

Bone metastases from tumors result in RANKL upregulation, inducing the stimulation of excessive bone resorption, which can lead to SREs (193). Denosumab has been approved on the basis of a high proportion of patients with solid tumor bone metastases or multiple myeloma achieving almost complete osteoclast inhibition with denosumab (121). The encouraging preclinical trials have provided the evidence for the role of denosumab in affecting the SREs of patients with bone metastases from tumors. The endpoints in clinical trials have included SRE incidence, time to first SRE, or bone marker changes (194, 195). In these trials, those who were treated with denosumab had lower biochemical markers of bone turnover (such as urinary Ntelopeptide, uNTX) and received a significant improvement in median time to first SRE in breast cancer and castration-resistant prostate cancer patients with metastatic bone disease (196–198). Other studies indicated the result that when breast cancer and prostate cancer patients were excluded, denosumab used in solid tumor and multiple myeloma was also superior to zoledronic acid (194). Denosumab, which can determine the course of a malignancy and potentially prevent metastatic outgrowth, provides one potential strategy for metastasis prevention. In men with nonmetastatic castration-resistant prostate cancer, denosumab increased the median bone metastasis-free survival by 4.2 months, and the occurrence of the first bone metastasis event was delayed using denosumab compared with placebo (199). However, no statistically superior bone metastasis-free survival was observed in high-risk early breast cancer with denosumab in the D-CARE study, which was an international, double-blind, randomized, placebo-controlled, phase 3 study conducted at 389 centers in 39 countries in women with early-stage breast cancer at moderate to high risk of disease recurrence (181). On the other hand, in the ABCSG-18 study including 3,425 postmenopausal women with estrogen receptor-positive or progesterone receptor-positive early breast cancer, adjuvant denosumab therapy delayed the disease recurrence in postmenopausal patients with early-stage lower-risk breast cancer (180). These results suggested that the role of denosumab to delay or inhibit the occurrence of bone metastasis in solid tumors requires further investigation to provide better information and more details about its biological effect (**Figure 3**).

## Denosumab in Combination With ICI

The randomized clinical trials about the efficacy of therapy using combinations of denosumab and immune checkpoint inhibitors (ICIs) targeting CTLA4, programmed cell death protein 1 (PD1), or programmed cell death 1 ligand 1 (PDL1) have not been prospectively unfolded in tumor bone metastasis patients. A few retrospective case series and case reports in bone metastatic melanoma have reported the synergism of ICI with denosumab. In case series including 10 patients with metastatic melanoma, the objective response rate was 60% and the disease control rate was 80% treated with denosumab and ICI (predominantly antiPD1 mAbs with or without antiCTLA4 mAbs), with the average treatment time of 9.8 months (200). Following these case studies, combination therapy targeting CTLA4 and RANKL provides more effective treatment inhibiting metastatic activities than either one of the agents alone in various mouse models (158). In 2016, a case of a melanoma patient, with aggressive and symptomatic bone metastases, was concomitantly treated with denosumab and anti-CTLA-4 antibody ipilimumab and was demonstrated to have a dramatic response and was alive at 62 weeks (177). Whether the combined effect of anti-CTLA-4 and anti-RANKL is successful is dependent on the lymphocytes’ action, as treatment was completely ineffective in mice lacking all lymphocytes and in mice depleted of natural killer cells (177). In another study, inhibition of RANKL has been shown to augment the antimetastatic efficacy of anti-PD-1/PD-L1 monoclonal antibodies in the prostate, colorectal cancer, and melanoma cell lines in mouse models (178). Mechanisms underlying the synergistic effect of anti-RANKL and ICIs remain unclear. Ahern et al. provide interesting possibilities that the optimal combination depended on the presence of activating Fc receptors and lymphocytes (particularly natural killer and CD8+ T cells) (201). The data on anti-RANKL/anti-PD1/anti-CTLA4 combination therapy showed promising results in a higher proportion of tumor-infiltrating CD4+ and CD8+ T cells that can produce both IFNγ and TNFα after anti-RANKL treatment. Such TME changes can increase ICB response through the attenuation of PD1 expression (178). Dougall et al. further underline the role of CD8+ T cells and IFNγ in enhancing PD-1 antibody efficacy. RANKL/RANK blockade can reverse the role of PD-1 blockade in CD8+ T-cell dysfunction and improve the antitumor efficacy of PD-1 antibody. However, the effect was abrogated by the depletion of CD8+ T cells or neutralization of IFNγ, suggesting major roles for RANKL/RANK blockade in an immune cell-dependent manner in the ICI response (202).

## Conclusion and Perspectives

Advances in research about the early events of tumor seeding, dormancy, and local niche remodeling have provided new insights into the role of the bone microenvironment to facilitate the growth of metastatic tumors in the bone. The studies discussed above illustrate how the changes of the immune microenvironment contribute to bone metastases, providing a highly modular platform potentially applicable to a broad range of cancer bone metastases. New molecules targeting bone metastases should combine with immune modulation in order to achieve effective eradication of metastatic lesions with minimal side effects.

In the past decades, many studies have uncovered the biological network associated with RANKL–RANK in immune systems, the development of lymphoid organs, bone metastasis regulation, and the initiation and progression of sex hormone-driven mammary cancer. Now that RANKL inhibitors to block osteoclast differentiation could be effectively used in treatments of osteoporosis, bone loss, and bone metastasis, the knowledge about how to modulate RANK signaling is needed to be further excavated and long-term clinical studies are needed to estimate whether the combined use of inhibitors with conventional therapies or ICIs leads to optimal treatment for bone metastasis patients.

## Author Contributions

BL and PW provided direction and guidance throughout the preparation of this manuscript. JJ and HW conducted the literature review and drafted the manuscript. PZ and WX reviewed the manuscript and made significant revisions on the drafts. All authors read and approved the final manuscript.

## Funding

This work was supported by grants from the National Natural Science Foundation of China (81902733), the Shanghai Science and Technology Committee (Grant No. 17411950300, 17411950301), and the Sailing Plan Project of Shanghai Municipal Commission of Science and Technology (19YF1448100).

## Conflict of Interest

The authors declare that the research was conducted in the absence of any commercial or financial relationships that could be construed as a potential conflict of interest.

## Publisher’s Note

All claims expressed in this article are solely those of the authors and do not necessarily represent those of their affiliated organizations, or those of the publisher, the editors and the reviewers. Any product that may be evaluated in this article, or claim that may be made by its manufacturer, is not guaranteed or endorsed by the publisher.
